# Finding optimal threshold for correction error reads in DNA assembling

**DOI:** 10.1186/1471-2105-10-S1-S15

**Published:** 2009-01-30

**Authors:** Francis YL Chin, Henry CM Leung, Wei-Lin Li, Siu-Ming Yiu

**Affiliations:** 1 Department of Computer Science, The University of Hong Kong, Pokfulam, Hong Kong, PRChina; 2 the State Key Laboratory of Computer Science, Institute of software, Chinese Academy of Sciences, 100190, Beijing, PR China

## Abstract

**Background:**

DNA assembling is the problem of determining the nucleotide sequence of a genome from its substrings, called *reads*. In the experiments, there may be some errors on the reads which affect the performance of the DNA assembly algorithms. Existing algorithms, e.g. ECINDEL and SRCorr, correct the error reads by considering the number of times each length-*k *substring of the reads appear in the input. They treat those length-*k *substrings appear at least *M *times as correct substring and correct the error reads based on these substrings. However, since the threshold *M *is chosen without any solid theoretical analysis, these algorithms cannot guarantee their performances on error correction.

**Results:**

In this paper, we propose a method to calculate the probabilities of false positive and false negative when determining whether a length-*k *substring is correct using threshold *M*. Based on this *optimal *threshold *M *that minimizes the total errors (false positives and false negatives). Experimental results on both real data and simulated data showed that our calculation is correct and we can reduce the total error substrings by 77.6% and 65.1% when compared to ECINDEL and SRCorr respectively.

**Conclusion:**

We introduced a method to calculate the probability of false positives and false negatives of the length-*k *substring using different thresholds. Based on this calculation, we found the optimal threshold to minimize the total error of false positive plus false negative.

## Background

DNA *assembling *is the problem of determining the nucleotide sequence of a genome from its substrings, called *reads*. Since DNA assembling is the first step in bioinformatics research, there are many different technologies, e.g. BAC-by-BAC approach [1], Sanger technique [2], for getting reads from a genome and there are many assembly algorithms [3-5] for solving the DNA assembling problem. In recent years, there is a technology breakthrough on getting reads from genomes. While the traditional technologies produce long reads (600–700 bp) with low coverage (each nucleotide is covered by 10 different reads) and low error rate, the Next Generation Sequencing (NGS) technologies, e.g. Solexa [6], Illumina [7], can produce short reads (25–300 bp) with high coverage (each nucleotide is covered by > 30 different reads) and high error rate using much less time and cost. Theoretically, we can determine the sequence of a genome in much shorter time and lower cost using the NGS technologies. However, many existing DNA assembling algorithms [8-10] were designed for traditional technologies which can handle reads with low error rate only and many new algorithms [11-14] designed for the NGS technologies assume the input reads are error free. Correcting errors in reads becomes an important problem for DNA assembling [15].

Since the NGS technology produces reads with high coverage, a read may be sampled several times in the genome. Under the assumption that an error read is unlikely to be sampled several time, Sundquist et al. [16] designed an algorithm called SHRAP which corrects the error reads by considering the number of times a read being sampled. If a read is sampled more than *M *times, for some predefined threshold *M*, it is considered as a correct read, otherwise, an error read which will not be used in the assembly step.

However, since the reads are randomly sampled from the genome, some correct reads may be sampled less (<*M *times) than the others, it is difficult to determine the threshold *M *to minimize the number of false negatives (increases with *M*) and the number of false positives (decreases with *M*). Besides, many reads with only one or two errors are wasted and will not be considered in the assembly step.

In order to consider reads with only one or two errors in the assembly step (which will increase the performance of the algorithm), Chaisson et al. [17] proposed another approach, called ECINDEL, to correct the errors in reads. Instead of considering the number of times a read being sampled, they considered the number of times each length-*k *substrings, called *k*-tuple, being sampled. A *k*-tuple is treated as correct if and only if it is sampled at least *M *times. By reducing the value of *k*, a higher threshold *M *can be set (compared to SHRAP) such that both the false positives and false negatives are small. Besides, error reads can be corrected by replacing some nucleotides in the reads such that all length-*k *substrings in the reads are correct *k*-tuples. Although this method seems nice, the value of *k *cannot be set to an arbitrary small number, e.g. when *k *= 1, we know that all 1-tuple, 'A', 'C', 'G' and 'T' are correct and we cannot use this information to correct the error reads. Thus there is still a problem of how to set the optimal thresholds *k *and *M*.

Wong et al. [15] designed another algorithm, called SRCorr, which improves ECINDEL by considering multiple *k *and *M*. Instead of considering only one pair of thresholds *k *and *M*, several sets of correct *k*-tuples with different lengths are determined and used to correct the error reads. Although some improvements have been made on correcting error reads, it is still difficult to set the thresholds.

In this paper, (1) we propose a method to calculate the probabilities of false positive and false negative for different substring lengths *k *and thresholds *M *in a data set. Experimental results show that the calculated probabilities match with the real data and simulated data. (2) Based on this calculation, we calculate the *optimal M *(minimizing the total errors = false positives + false negatives) for each substring length *k*. By using the optimal threshold *M*, the total errors can be reduced by 77.6% and 65.1% when compared to ECINDEL [17] and SRCorr [15] respectively.

## Results and Discussion

When the hidden genome *G *is known, we can count the number of true positives TP (*k*-tuples occur in *G *which are sampled at least *M *times), false positives FP (*k*-tuples do not occur in *G *which are sampled at least *M *times) and false negatives FN (*k*-tuples occur in *G *which are sampled less than *M *times) for each threshold *M*. Therefore, we can find the optimal threshold *M *that minimizes the total errors FP + FN.

However, when solving the DNA assembling problem, the genome *G *is unknown. Both ECINDEL [17] and SRCorr [15] do not have a sound theoretical analysis on how to set the threshold *M*. When the number of sampled reads is large, even the incorrect *k*-tuples are sampled *M *times or more, these algorithms have many false positives. When the number of sampled reads is small, even the correct *k*-tuples are sampled less than *M *times, these algorithms have many false negatives. Instead of using an arbitrary threshold *M*, we calculate the expected number of true positives, false positives and false negatives according to the equations described in the Methods Section. By considering the optimal threshold *M *that minimizes the expected false positives plus false negatives (FP + FN), we can get a set of *k*-tuples with the minimum expected number of errors. In this paper, we will perform experiments on both real experimental data and simulated data. The experimental results show that (1) the expected number true positives, false positives and false negatives match with the real data. Therefore, the optimal threshold *M *calculated by us minimizes the total errors (FP + FN). (2) By using the optimal threshold *M *calculated by us, the total errors reduced by 77.6% and 65.1% when compared to ECINDEL and SRCorr respectively.

### Experimental results on real data

We performed experiments on a real data set from the human genome. The hidden genome is a subregion of the human genome of length 173427, length-35 reads are sampled from the genome using Solexa [6] techniques.

**Figure 1:**
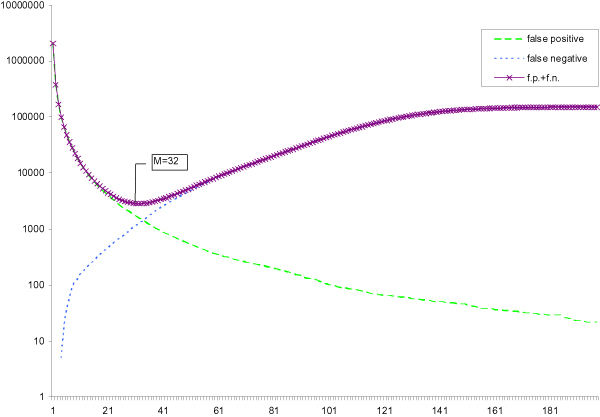
**k = 15**. Number of false positives, false negatives and their sum of 15-tuples from real data versus multiplicity.

**Figure 2:**
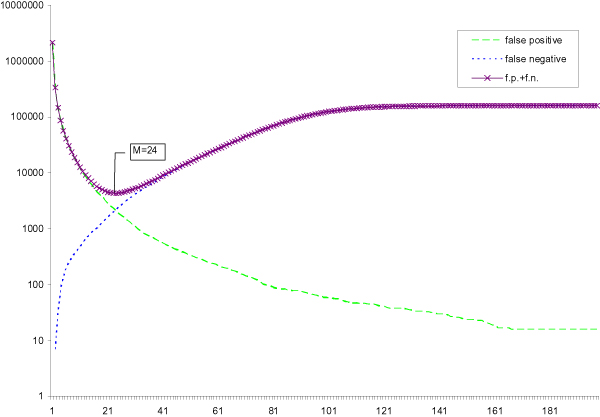
**k = 20**. Number of false positives, false negatives and their sum of 20-tuples from real data versus multiplicity.

Figures 1 and 2 show the number of false positives and false negatives for different threshold *M *on this data set when the substring length *k *are 15 and 20 respectively. Since the number of false positives decreases with *M *and the number of false negatives increases with *M*, the total errors (FP + FN) is a U-shape curve. The minimum point of this curve represents the optimal threshold *M *that minimizes the total errors. Besides, the optimal M* increases when the length of the *k*-tuple decreases. For example, the optimal threshold M* for 15-tuples is 32 which is larger than the optimal threshold M* for 20-tuples (M* = 24). According to the equations in the Method Section, we can calculate the expected number of false positives and false negatives. Thus, we can find the threshold *M *with the minimum expected number of errors. We find that the threshold *M *is exactly the same as the optimal threshold M*

We compared the number of false positives and false negatives of ECINDEL and SRCorr with our algorithm. In the experiment, we used *k *= 15 which is the default parameter of SRCorr. Since SRCorr uses a range of substring length *k*, when comparing the performance on *k*-tuples, we considered the 15-tuples of SRCorr only. When comparing the performance on reads, SRCorr runs with multiple *k *and *M *to correct errors on reads. Tables 1 and 2 show the performance of the algorithms on 15-tuples and reads respectively.

As described in Table 1, ECINDEL produces a set of 15-tuples with 12670 errors (FP + FN). By considering multiple thresholds, SRCorr reduces the number of errors to 8140. Since the converge of this dataset is high, instead of using a small threshold *M *(12 and 15), we calculated an optimal threshold M* = 32 and reduced the number of errors to 2839. Therefore, the number of errors were reduced by 77.6% and 65.1% when compared with ECINDEL and SRCorr respectively. With a better set of 15-tuples than ECINDEL and SRCorr, we corrected the reads such that the total errors reduced to 33477 when compared with ECINDEL(192533) and SRCorr(212287) respectively. Note that when considering the corrected reads, we have less false positives and less false negatives than these two algorithms.

**Table 1:** Comparison of 15-tuples on real data set

	M	# of false positives	# of false negatives	total errors
ECINDEL	12	12494	176	12670
SRCorr	15	7890	250	8140
Our Algorithm	32	1583	1256	2839

**Table 2:** Comparison of corrected reads on real data set

	M	# of false positives	# of false negatives	total errors
ECINDEL	12	19334	173199	192533
SRCorr	15	181628	30659	212287
Our Algorithm	32	30039	3438	33477

### Experimental results on simulated data

In this section, we compared the performance of ECINDEL, SRCorr and our algorithm on simulated data. The simulated data was generated as follows: We generated a length-*g *genome sequence *G *with equal occurrence probability of each nucleotide (1/4). *n *length-35 reads were sampled from *G *with equal probabilities. Each nucleotide in each read could mutate to another nucleotide with probability *p*_*err*_. The probability that a nucleotide mutates to each of the other nucleotide is the same (1/3). The *n *length-35 reads (after mutation) were considered as input for the algorithms. Similarly with the experiments on real data, we set the default parameter *k *= 15 of SRCorr when comparing the *k*-tuples. When comparing the performance on reads correction, SRCorr runs with multiple *k *and *M*.

Tables 3 and 4 show the performances of the algorithms on 15-tuples and reads respectively when *g *= 79745, *n *= 220000 and *p*_*err *_= 4%.

**Table 3:** Comparison of 15-tuples on simulated data (coverage = 2.75×)

	M	# of false positives	# of false negatives	total errors
ECINDEL	12	4	18	22
SRCorr	4	5264	5	5269
Our Algorithm	11	5	13	18

**Table 4:** Comparison of corrected reads on simulated data (coverage = 2.75×)

	M	# of false positives	# of false negatives	total errors
ECINDEL	12	46459	49204	95663
SRCorr	4	53036	31699	84735
Our Algorithm	11	308	8564	8872

Since there were relatively fewer reads being sampled (coverage = 2.75×) in this data set, SRCorr applied a small threshold *M *= 4 for the 15-tuples. As SRCorr chose this threshold without much analysis, the threshold selected was too small such that there were many false positives and the total errors was 5269 for the 15-tuples. ECINDEL applied a fix threshold *M *= 12 and the total errors was 22. Based on the optimal threshold M* = 11 we derived, we produced a set of 15-tuples with the fewest number of errors (18 errors). Similarly for the real data set, since we have derived a set of 15-tuples with less errors than ECINDEL and SRCorr, we could correct more error reads than ECINDEL and SRCorr (8872 errors instead of 95663 errors and 84735 errors). The corrected reads produced by us had less false positives and false negatives than ECINDEL. Since SRCorr applied a small threshold, 75863 more false positive reads were introduced when compared with our algorithm.

Table 5 and 6 show the performances of the algorithms on 15-tuples and reads respectively when *g *= 79745, *n *= 1000000 and *p*_*err *_= 4%.

**Table 5:** Comparison of *k*-tuples on simulated data (coverage = 12.53×)

	M	# of false positives	# of false negatives	total errors
ECINDEL	12	1699	4	1703
SRCorr	15	1011	5	1016
Our Algorithm	52	3	15	18

**Table 6:** Comparison of corrected reads on simulated data (coverage = 12.53×)

	M	# of false positives	# of false negatives	total errors
ECINDEL	12	132850	30338	163188
SRCorr	15	102288	1512	103800
Our Algorithm	52	1216	49	1265

When compared to the previous set of simulated data, we had more sampled reads in this data set (coverage = 12.53×). Since ECINDEL and SRCorr applied a small threshold (12 and 15) for determining correct 15-tuples, they had many errors (1703 and 1016). Instead of using a small threshold, we arrived at an optimal threshold M* = 52 which cound determine the correct 15-tuples with 18 errors only. With a set of 15-tuples with less errors, we could correct the errors in reads better than ECINDEL and SRCorr and produced a set of reads with 1265 errors, much less than ECINDEL and SRCorr (163188 and 103800 errors respectively), and in terms of the number of false positives and false negatives; both of them were less than ECINDEL's and SRCorr's results.

## Conclusion

We have studied the problem of correcting error reads in DNA assembling. We introduced a method to calculate the probability of false positives and false negatives of the *k*-tuples using different thresholds *M*. Based on this calculation, we found the optimal threshold M* that minimizes the total error (FP + FN). Our calculation can also be extended to total errors with different weightings of FP and FN. Our algorithm, which uses optimal threshold M* to correct error reads, performs better than the popular algorithms ECINDEL and SRCorr.

In the real biological data, we might not be able to remove all the false positives and false negatives by a fixed threshold *M*. It is mainly because the probability of each read being sampled is not the same in real experiment. This probability depends on the patterns of the reads, the positions of the reads in the genome and the adjacent reads. A better model might be needed to determine whether a *k*-tuple is correct (instead of using a fixed threshold *M*) and to correct more error reads.

## Methods

In this section, we will first describe Chaisson et al.'s [17] approach, called ECINDEL, for correcting error reads. Sundquist et al.'s [16] approach is a special case of ECINDEL by setting *k *equals read length and Wong et al.'s [15] approach is a general case of ECINDEL by considering multiple *k*. Then we will describe how to calculate the probability of true positive, false positive and false negative for determining whether a *k*-tuple is correct by threshold *M*. Based on this calculation, we will describe how to determine the optimal threshold M* for Chaisson et al.'s, Sundquist et al.'s and Wong et al.'s approach.

### ECINDEL algorithm

Given a set of reads *R *from a hidden genome *G*, ECINDEL determines a set *G*_*k *_of length-*k *substrings, *k*-*tuples*, which appear in more than *M *reads in *R*. ECINDEL considers all *k*-tuples in *G*_*k *_correct (are substrings of *G*) and all *k*-tuples not in *G*_*k *_incorrect (are not substrings of *G*). Under the assumption that all *k*-tuples of a correct read are correct, ECINDEL considers reads with all its *k*-tuples in *G*_*k *_as correct reads. For those reads *s *with *k*-tuples not in *G*_*k*_, ECINDEL tries to correct errors in *s *by modifiying *s *to another read *s' *with the minimum number of operations (edit distance) such that all *k*-tuples in *s' *are in *G*_*k*_. As you can see, the performance of this algorithm depends on the quality of the set *G*_*k*_. If there are many false negatives(correct *k*-tuple not in *G*_*k*_), ECINDEL will modifiy the correct reads to error reads or another correct reads which will decrease the number of correct reads in the input. If there are many false positives (incorrect *k*-tuple in *G*_*k*_), ECINDEL will treat some error reads as correct reads which will affect the performance of the assembly algorithms.

In order to calculate the probabilities of false positive and false negative, we assume the reads *R *sampled from *G *are generated as follows: Let *G *be a genome sequence of length *g *and we sample *n *length-*l *substrings (reads) (set *R*) from *G *independently. Every position is uniformly sampled from *G *with the same probability 1/(*g *- *l *+ 1) (The same position can be sampled more than once). Each nucleotide in each read in *R *may be erroneous with probability *p*_*err*_. We consider all length-*k *substrings (*k*-tuples) of every length-*l *read in *R*, *k *= l, as input.

Given a *k*-tuple *T*, let

• *T*_*t *_be a variant of *T *with exactly *t *mismatches (*t *= 0, ..., *k*, *T*_0 _= *T*).

• *Y*_*r *_be the event that *T *appears exactly *r *times in *R*.

• *X*_*t *_be the event that *T*_*t *_is a part of the reference sequence.

Assume we treat all *k*-tuples *T *with sample numbers *r *≥ *M *as substrings of *G*, we want to calculate the probabilities of *T *being a true positive *Pr*(*X*_0_|∪_*r*≥*M *_*Y*_*r*_), false positive 1 - *Pr*(*X*_0_|∪_*r*≥*M *_*Y*_*r*_) and false negative *Pr*(*X*_0_|∪_*r *<*M *_*Y*_*r*_)).

### Probabilities of false positive and false negative

In this section, we will calculate the probabilities of *T *being a true positive *Pr*(*X*_0_|∪_*r*≥*M *_*Y*_*r*_), false positive 1- *Pr*(*X*_0_|∪_*r *> *M *_*Y*_*r*_) and false negative *Pr*(*X*_0_|∪_*r *<*M *_*Y*_*r*_)) assuming that *T *is a *k*-tuple where sample number ≥ threshold *M*. Since the calculation of these probabilities depends on the value of *Pr*(*Y*_*r*_), the probability that *T *appears exactly *r *times in *R*, we will first describe how to calculate *Pr*(*Y*_*r*_). Then we will describe how to calculate the true positive, false positive and false negative based on *Pr*(*Y*_*r*_). Assume *k*-tuple *T *is sampled *r *times in the set of reads *R*, the *r *samples of *T *coming from *r *reads (length-*l *substrings in *G*) appear in different positions of *G *and multiple copies of *T *may be sampled from the same position. Copies of *T *are sampled at different positions either because (1) a *k*-tuple *T *= *T*_0 _occurs in one of these positions of *G *or (2) a variant *T*_*t *_of *T *occurs in one of these positions of *G *and *T *is sampled because of error. When *t *is small (e.g. *t *= 0, 1, 2), the probability that *T *being sampled from these positions will be considered. When *t *is large (e.g. *t *= *k*), the probability that *T *being sampled from these positions is low and can be ignored. We first calculate the probability *p*_*occ*_(*g*, *t*) that a variant *T*_*t *_of *T*, *t *= 0, ..., *k*, appears in a particular position of a length-*g *genome sequence *G*. Then we calculate the probability *p*_*sam*_(*g*, *s'*) that a particular position is sampled *s' *times and the probability *p*_*count*_(*g*, *s'*, *r'*) that *r' *out of these *s' *samples are *T *. By considering all positions of *G*, we can calculate the probability *Pr*(*Y*_*r*_) that *T *appears exactly *r *times in *R*.

Given a length-*g *random genome *G *with each nucleotide having the same occurrence probability (1/4), the probability that a variant *T*_*t *_of *T *occurs at a particular position *i *of *G*, i.e. *G *[*i*...*i *+ *k *- 1] is a variant *T*_*t *_of *T*, is

$$p_{occ}(g,t)=\frac{3^{t}\left(\begin{array}{c} k\\ t \end{array}\right)}{4^{k}}$$

Genome sequences, especially the non-coding sequences, are highly heterogeneous in composition. The i.i.d. model cannot reflect the real situation of the genome sequences well. On the other hand, genome *G *is generated by other models, e.g. Markov Chain, *p*_*occ*_(*g*, *t*) can also be calculated easily. However, this part has little effect on the overall result, as the number can be cut in the later calculation.

Let *T*_*t *_be a *k*-length substring obtained at position *i *of *G*. Since only those reads containing the substring from position *i *to *i *- *k *+ 1 can contribute *T*_*t*_, copy of *T*_*t *_can be obtained from at most *l *- *k *+ 1 different reads, exactly *l *- *k *+ 1 reads in most cases when *l *- *k *+ 1 ≤ *i *≤ *g *- *l *+ 1. When *g *>> *l*, the probability that *T*_*t *_is sampled *s' *times at can be approximated by

$$p_{sam}(g,s^{\prime })=\left(\begin{array}{c} n\\ s^{\prime } \end{array}\right){\left(\frac{l-k+1}{g}\right)}^{s^{\prime }}{\left(1-\frac{l-k+1}{g}\right)}^{n-s^{\prime }}$$

When *g *is large, *p*_*sam*_(*g*, *s'*) can be approximated by the normal distribution with mean equals

$$n\left(\frac{l-k+1}{g}\right)$$

and variance equals

$$n\left(\frac{l-k+1}{g}\right)\left(1-\frac{l-k+1}{g}\right)$$

In some real experimental data where each position is not sampled with the same probability, we may estimate the mean and variance of *p*_*sam*_(*g*, *s'*) from training data.

When a read with variant *T*_*t *_of *T *is sampled, the probability *p*_*t *_that we get the *k*-tuple *T *instead of *T*_*t *_as input because of error is

$$p_{t}={\left(1-p_{err}\right)}^{k-t}{\left(\frac{p_{err}}{3}\right)}^{t}$$

where *p*_*err *_is the probability of single nucleotide error occurrence. Note that as *p*_*err *_is usually very small, *p*_*t *_can be ignored and assumed zero when *t *≥ 3 in practice. Here we assume when there is error, the occurrence probability of each nucleotide (three possible nucleotides) is the same. Similar as *p*_*occ*_(*g*, *t*), the formula for *p*_*t *_can be modified when genome *G *is generated by other models. The probability that the *k*-tuple *G *[*i*...*i *+ *k *- 1] is sampled *s' *times and *r'*(*r' *≤ *s'*) of them is *T *because of errors is

$$p_{count}(g,s^{\prime },r^{\prime })=\sum_{t^{\prime }=0}^{t}p_{occ}(g,t^{\prime })p_{sam}(g,s^{\prime })\left(\left(\begin{array}{c} s^{\prime }\\ r^{\prime } \end{array}\right)p_{t^{\prime }}^{r^{\prime }}{\left(1-p_{t^{\prime }}\right)}^{s^{\prime }-r^{\prime }}\right)$$

where *G *[*i*...*i *+ *k *- 1] = *T*_*t*'_, 0 ≤ *t' *≤ *t*. In order to get *r *samples of *T *from the *n *reads, *d*(*d *= 1, ..., *r*) variants of *T *appear in different positions of *G *and *r*_*j *_samples of *T *are getting from the *j*-th variants such that Σ*r*_*j *_= *r*. Therefore, the probability that *T *appears exactly *r *times in the input is approximately

(1) $$\begin{array}{c} P_{r}(Y_{r})\\ =\sum_{d=1}^{r}\sum_{r\leq \sum s_{j}\leq n(l-k+1)}\sum_{r_{j}\leq s_{j},\sum r_{j}=r}\frac{(n-k+1)!}{d!}\prod_{s_{j}}p_{count}(g,s_{j},r_{j})\\ {\left(\sum_{t=1,...,k}p_{occ}(g,t)(1-p_{t})\right)}^{n(l-k+1)-\sum s_{j}} \end{array}$$

Equation (1) is an approximation because we have not considered the interdependence of the positions of the *d *variants of *T *and the samples in the remaining positions. This approximation is fine when *g *>> *d *and *n *is large, which is valid for most experimental data.

Once we calculate *Pr*(*Y*_*r*_), we can calculate the probability of true positive as follows:

$$\begin{array}{l} Pr(X_{0}|\cup_{r\geq M}Y_{r})\\ =\frac{Pr(\cup_{r\geq M}(Y_{r}\cap X_{0})}{P(\cup_{r\geq M}Y_{r})}\\ =\frac{\sum_{r=M}^{n}Pr(Y_{r}\cap X_{0})}{\sum_{r=M}^{n}P(Y_{r})}\\ =\frac{\sum_{r=M}^{n}\left(1-Pr(Y_{r}\cap \neg X_{0})\right)}{\sum_{r=M}^{n}P(Y_{r})}\\ =\frac{\sum_{r=M}^{n}\left(1-Pr(Y_{r}|\neg X_{0})Pr(\neg X_{0})\right)}{\sum_{r=M}^{n}P(Y_{r})} \end{array}$$

*P*(*Y*_*r*_) can be calculated from Equation (1). *Pr*(¬ *X*_0_) = (1 - *p*_*occ*_(*g*, 0))^*n*-*k*+1 ^and *Pr*(*Y*_*r*_|¬ *X*_0_) can be calculated from Equation (1) by considering that the probability that *T *appearing in *G *is zero, i.e. set ${p^{\prime }}_{occ}$ (*g*, 0) = 0 and ${p^{\prime }}_{occ}$ (*g, t*) = *p*_*occ *_(*g*, *t*)/(1 - *p*_*occ*_(*g*, 0)).

The probability of false positive one minus the probability of true positive

$$\begin{array}{l} 1-Pr(X_{0}|\cup_{r\geq M}Y_{r})\\ =1-\frac{\sum_{r=M}^{n}\left(1-Pr(Y_{r}|\neg X_{0})Pr(\neg X_{0})\right)}{\sum_{r=M}^{n}P(Y_{r})} \end{array}$$

The probability of false negative can also be calculated similarly:

$$\begin{array}{l} Pr(X_{0}|\cup_{r<M}Y_{r})\\ =\frac{Pr(\cup_{r<M}(Y_{r}\cap X_{0})}{P(\cup_{r<M}Y_{r})}\\ =\frac{\sum_{r=0}^{M-1}Pr(Y_{r}\cap X_{0})}{\sum_{r=0}^{M-1}P(Y_{r})}\\ =\frac{\sum_{r=0}^{M-1}\left(1-Pr(Y_{r}\cap \neg X_{0})\right)}{\sum_{r=0}^{M-1}P(Y_{r})}\\ =\frac{\sum_{r=0}^{M-1}\left(1-Pr(Y_{r}|\neg X_{0})Pr(\neg X_{0})\right)}{\sum_{r=0}^{M-1}P(Y_{r})} \end{array}$$

Since we only consider integer threshold *M*, once we calculate the probabilities of true positive, false positive and false negative for all possible thresholds *M *for a particular substring length *k*. We can then find the optimal threshold M* for that particular *k *which minimizes the total errors FP + FN or maximizes the total accuracy.

$$\sqrt{\frac{TP}{TP+FP}•\frac{TP}{TP+FN}}$$

## Competing interests

The authors declare that they have no competing interests.

## Authors' contributions

FC and SMY studied the read correction problem, suggested the main idea of calculating the optimal threshold M. HL and WLL worked out the details of calculation, worked out the experiments and drafted the manuscript. All authors read and approved the final manuscript.
